# Length of stay of hospitalized patients at tertiary psychiatry facilities in Uganda: the role of caregiver’s presence

**DOI:** 10.1007/s44192-022-00018-x

**Published:** 2022-07-04

**Authors:** Mark Mohan Kaggwa, Maria Sarah Najjuka, Claire Kesande, Novatus Nyemara, Moses Kule, Mohammed A. Mamum, Felix Bongomin, Scholastic Ashaba

**Affiliations:** 1 Department of Psychiatry, Faculty of Medicine, Mbarara University of Science and Technology, Mbarara, Uganda; 2 African Centre for Suicide Prevention and Research, Mbarara, Uganda; 3 Department of Psychiatry and Behavioural Neurosciences, McMaster University, Hamilton, Canada; 4 College of Health Sciences, Makerere University, kampala, Uganda; 5 Butabika National Referral and Teaching Mental Hospital, kampala, Uganda; 6 Department of Psychiatry, Mbarara Regional Referral Hospital, Mbarara, Uganda; 7 CHINTA Research Bangladesh, Dhaka, Savar, Bangladesh; 8 Department of Public Health and Informatics, Jahangirnagar University, Dhaka, Savar, Bangladesh; 9 Department of Medical Microbiology & Immunology, Faculty of Medicine, Gulu University, Gulu, Uganda

**Keywords:** Length of stay, Psychiatry inpatient, Caregiver, Mental health services, And Uganda

## Abstract

**Background:**

Whether the presence of caregivers during the hospital stay of patients with mental illness affects the length of hospital stay (LoS) remains inconclusive.

**Aims:**

(1) To determine the average LoS and the associated factors, and (2) to determine the role of caregivers’ presences during inpatient stay on LoS.

**Methods:**

We conducted a cross-sectional study in two hospitals in Uganda; one with caregivers and the other without caregivers between July to November 2020. Mann-Whitney U test was used to compare LoS in the two selected hospitals and linear regression was used to determine factors associated with LoS.

**Results:**

A total of 222 participants were enrolled, the majority were males (62.4%). Mean age was 36.3 (standard deviation (*SD*) = 13.1) years. The average LoS was 18.3 (*SD* = 22.3) days, with patients in a hospital without caregivers having a longer median LoS (i.e., (30 (interquartile range (IQR) = 30) vs. 7 (7) days; χ^2^ = 68.95, *p* < 0.001). The factors significantly associated a longer LoS among our study participants included; being admitted in a hospital without caregivers (adjusted coefficient [aCoef]: 14.88, 95% CI 7.98–21.79, *p* < 0.001), a diagnosis of schizophrenia (aCoef: 10.68, 95 %CI 5.53–15.83, *p* < 0.001), being separated or divorced (aCoef: 7.68, 95% CI 1.09–14.27, *p* = 0.023), and increase in money spent during the admission (aCoef: 0.14, 95% CI 0.09–0.18, *p* < 0.001).

**Conclusion:**

Patients with mental illness in southwestern Uganda have a short LoS (below 28 days), and the stay was much shorter for patients with fulltime caregivers. We recommend caregivers presence during patient’s hospital stay to reduce the LoS and minimize healthcare expenditure.

## Introduction

The average length of hospital stay (LoS) in mental health units is often used as an indicator of efficient health care service usage [1]. With other factors being constant, a short LoS reduces the cost of care by shifting care from inpatient to community which is less expensive [1]. On the other hand, a longer LoS is associated with more medical and/or psychiatric comorbidities, for example, nosocomial infections, depression, among others; thus, more medical costs and bills, worsening prognosis, creating a significantly higher burden on caregivers in addition to a higher burden on hospital and government resources [2–7]. Literature has reported variations in LoS depending on the location, shortest in the USA (10 days) [6, 8], longer in Japan at about 42 days [9], and longest in Korea (168 days) [1]. However, LoS is documented to range between 14 to 28 days among patients admitted in sub-Sahara African psychiatric facilities [3, 10–12]. In Uganda – where the present study was conducted, LoS has been reported at about 21 days for a National Referral Mental Hospital, and about 14 days for psychiatric units within regional referral hospitals [13].

Despite no previous studies done in Uganda to assess factors associated with LoS; previous studies in sub-Sahara Africa have identified a number of factors, including being unmarried, unemployed, old age, type of mental illness (e.g., schizophrenia, bipolar affective disorder (BAD), mental retardation, and seizure disorder), as well as comorbid medical conditions (e.g., HIV, epilepsy, and hepatitis), extrapyramidal side effects (EPS), substance use, and previous hospitalizations [3, 10–12, 14, 15]. Additionally, admission to a teaching or public hospital, higher density of psychiatric beds, and non-compliance to medicines have been documented to increase the duration of hospital stay [3, 9, 14]. On the other hand, improved medical technology such as electroconvulsive therapy (ECT), private hospitals, and human resources presence have been associated with reduced LoS [2, 3, 9, 12, 14].

On the contrary, there have been mixed findings on the impact of caregivers on LoS [4]. Some studies have found that caregivers’ presence increases the LoS [16, 17], while others have shown no particular impact [4, 18]. Those studies indicating the presence of caregivers to be associated with longer LoS have attributed this to the various burdens faced by the caregivers divided into subjective and objective components [4, 19]. Subjective care burden, including the personal appraisal of the caring role, including attitude and emotions, for example, caregivers’ stress or affiliated stigma, have increased LoS in hospitals [7, 20]. On the other hand, objective care burdens, including observable or tangible costs to caregivers such as monetary costs, disruption of daily routine, or time, have been noted to lengthen hospital stay among patients with mental health illnesses [21]. Additionally, caregivers have various roles during the period of admission of patients, such as emotional support, encouraging and motivating the patient, assistance with practical tasks like managing finances, transportation, feeding, and other activity of daily living (ADL), for example, personal hygiene, and a decision on whether a patient needs admission or discharge [19, 22, 23]; might influence the recovery of patients hence shortening the length of hospital stay.

In Uganda, mental health facilities have different policies on caregivers’ involvement in a patient’s hospital stay. For example, the only National Referral Mental Hospital, which is public mental health hospital and Kampala International University Teaching (KIUTH), a private hospital with a mental health unit, do not admit patients with mental illness with their caregivers, while in all regional referral hospitals in Uganda, patients are attended to by their caregivers. Moreover, the insufficient allocation (1%) of the national health budget to mental health in Uganda [24], is lower than in most countries, whose medium percentage allocation is 5.1%, 2.4%, and 1.9% in high-income, upper-middle, and lower-middle-income countries respectively have led to poor infrastructure and services [25]. Evidently, with this low budget, hospitals in Uganda are unable to afford to accommodate both patients and their caretakers in terms of feeding and lodging, and inpatient psychiatric beds are fewer (1.83 beds per 100,000 population) [24] compared to elsewhere in the world, estimated at 1.4 to 7.04 per 100,000 patients [26]. Despite this, the rate of admissions to mental hospitals in Uganda is high at 39.3 per 100,000 people and is increasing due to the increasing incidence of mental illness [26]. Regional referral hospitals allow caregivers to bridge the gap by providing food, security, and financial assistance, such as buying the prescribed drugs that are not on the national essential drug list [13, 27]. Therefore, reducing the LoS for patients by clinicians might improve care and reduce the health system costs, caregivers’ burden, and crowding of patients in the hospitals [4]. No study has been conducted to identify the factors associated with the length of hospitalization among patients with mental illness in Uganda. Therefore, the aims of this study are: (i) to determine the average LoS and the associated factors, and (ii) to determine the role of caregivers’ presences during inpatient stay on LoS.

## Methods

### Study design and setting

A cross-sectional analytical study was conducted at Mbarara Regional Referral Hospital (MRRH) and KIUTH psychiatry units between July and November 2020. The two hospitals are similar in that they all have a similar number of stationed psychiatrists, psychiatry resident students, nurses, social workers, and counselors. In addition, both hospitals do not admit forensic patients. However, KIUTH is a private hospital and does not always admit patients with their caregivers. MRRH has 36 beds while KIUTH has 50 beds, but the number of admissions in MRRH was twice that in KIUTH in 2019 (based on unpublished hospital records, i.e., on average, 14 [MRRH] vs. 7 [KIUTH] admissions per week).

### Study population and eligibility screening

We recruited patients aged 7 years and above, on the day of discharge. We excluded (i) patients readmitted during the time of the study, (ii) patients physically and mentally severely too ill to participate in the study or provide consent as determined by the attending clinician, and (iii) patients who were not able to communicate verbally and comprehend the contents of the consent document due to intellectual disability, cognitive disability, or being deaf and dumb.

### Sample size

Sample size was calculated using the sample formula for an infinite population with continuous outcomes based on the available means [28].

Equation: sample size estimation formula for the infinite population using available means

$${n}_{i}=2{\left(\frac{{z}_{\sigma }}{ES}\right)}^{2}$$

*where, n is the sample size, z is the z score for a confidence interval of 95%, ES is the estimated margin of error, *$\sigma$* is the standard deviation from previous studies.*

Using average LoS from studies done in some randomly chosen Sub-Sahara African countries; Ethiopia (28.7), Nigeria (22, 25 and 23.9), Malawi (22), and Uganda hospitals i.e. Mulago National referral Hospital (12), Butabika National Referral Hospital (21), Lira Regional Referral hospital (21), MRRH (14), Soroti Regional Referral Hospital (14), Mubende Regional Referral Hospital (14), Mbale Regional Referral Hospital (14), Hoima Regional Referral Hospital (14), Gulu Regional Referral Hospital (14), Moroto Regional Referral Hospital (14), Jinja Regional Referral hospital (14), Arua Regional Referral Hospital (21), Masaka Regional Referral Hospital (14), Kabale Regional Referral Hospital (14), and Fort Portal Regional Referral Hospital (14) [3, 10–13, 29], a standard deviation (SD) of 4.9 days was obtained.

Using ES of 1 day and z = 1.96

$${n=2\left(\frac{1.96x4.9}{1}\right)}^{2}$$

n = 184.5 = 185 participants.

We adjusted for non-respondents of approximately 20%; the total sample size is considered as 222 participants. With MRRH having twice more admissions than KIUTH, we consider a minimum of 148 for MRRH and 74 participants for KIUTH to have a similar representation of participants recruited within the same period.

### Sampling procedure

Participants were recruited using non-probability convenience sampling method at the point of their discharge from the hospital until the sample size for different study sites was reached, i.e., daily recruitment depended on the number of patients discharged on that day.

### Data collection

All information was collected by three (two at MRRH and one at KIUTH) research assistants (RA) trained in data collection methods, research ethics, questionnaire administration, in-depth interviews, and especially, how to ask sensitive questions. Potential respondents were approached on the day of discharge from the hospitals. Translated consent forms were provided to patients who were willing to participate in the study in the language of their choice (Runyakole/Rukiga). A unique identification number was assigned to make the data anonymous. For participants who could not read and write, RAs guided them to respond to all the questions appropriately. A special-colored paper was added to the patient’s hospital files and removed after the study period to limit enrolling the same patients.

### Questionnaire

A pretested questionnaire was used to extract patient’s data. The final questionnaire included socio-demographic information such as age, sex, address (rural vs. urban), marital status, employment, level of education, number of children, type of housing (private, public, homeless, or rental), and the patient is the family breadwinner. Information related to the hospitalization and medical conditions such as mode of admission (voluntary, involuntary); length of hospital stay, diagnosis (collected from patient hospital file), years with mental illness diagnosis, number of the previous admission due to mental illness, number of years since the last admission, comorbid medical condition such as diabetes, HIV, and hypertension, history of suicide attempts, patient history of pattern outpatient follow-ups, and history of being put in seclusion room during the admission, were also collected in this study.

### Ethical considerations

The study was conducted in accordance with the Declaration of Helsinki 2013. The study received ethical approval from the research ethics committee of Mbarara University of Science and Technology (#29/03-20). The directors of the hospitals granted permission to collect data from participants. All participants provided voluntary written informed consent at study enrollment. The individual below 18 years provided assent, and informed consent was obtained from their caregivers.

### Data analysis

All data were entered in STATA version 16 for cleaning and formal analysis. The mean LoS and median LoS were determined between the two sites. The Mann–Whitney U test was performed to check for statistical differences between the medians. For cases of outliers and comparisons of length of stay, data were represented on box and whisker plots. The outliers from each site were determined after calculating the Inter Quartile Range (IQR). A value was identified to be a potential outlier if it was more than (1.5 IQR) below the first quartile or more than (1.5) (IQR) above the third quartile. For descriptive statistics, mean, mode, median and standard deviations were used to summarize continuous variables, while proportions and percentages were used to summarize categorical variables. An independent sample t-test was used to compare numerical variables such as age at onset of disease and Pearson’s chi-square test to compare categorical data between the groups. Three separate linear regression modeling was used to assess the relative importance of factors identified as predictors of LoS among study participants in the total sample, those with caregivers and those without caregivers. All significant factors at bivariate linear regression were accessed for collinearity and only factors with a variance inflation factor (VIF) below three were included in multivariate linear regression. A *p* < 0.05 for the level of significance was considered at a 95% confidence interval.

## Results

### Characteristics of the participants

**Fig. 1:**
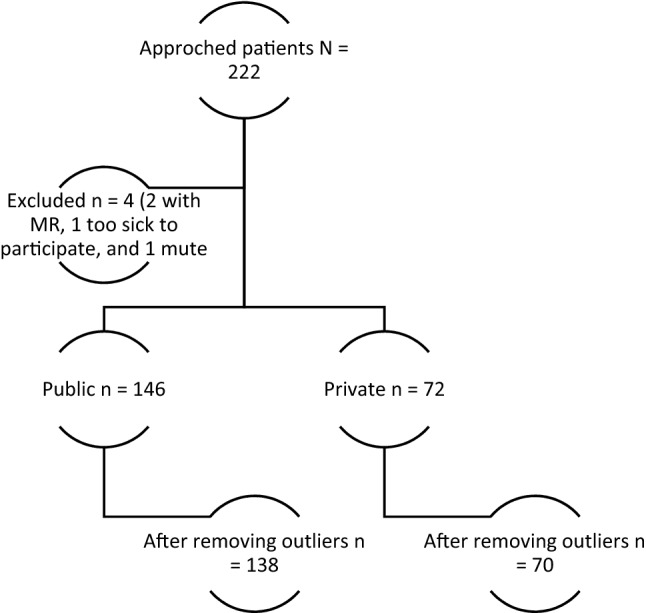
Flow chart showing how patients were included from the two study sites

A total of 226 participants were approached; four did not meet the inclusion criteria (Fig. 1). The average age of all participants was 36.3 ± 13.1 years, with patients with caregivers on average being 5 years older (37.90 vs. 33.15). Most (62.4%, n = 136) participants were males, and most were admitted to a hospital without caregivers than those with caregivers (76.4% vs. 55.6%, *p* = 0.003). Most of the participants were married or cohabiting (40.4%), employed (60.55%), dwelled in rural areas (72.5%), half of them were staying in their private homes, and 48% had attained education up to the primary level. Participants with caregivers had more children compared with those without caregivers, and the difference was statistically significant (2.90 vs. 1.96, *p* = 0.018). During admission, individuals without caregivers significantly spent more money than those with caregivers (74.22 USD vs. 20.23 USD, *p* < 0.001) (Table 1). Medical comorbidities were higher among patients without caregivers (45.8% vs. 31.5%, *p* = 0.038). However, patients with caregivers experienced more side effects as per patients’ hospital records (37.0% vs. 2.8%, *p* < 0.001). For details, see Table 2.

**Table 1:** Sociodemographic characteristics of the participants

Variable	N	With caregivers (n = 146)	Without caregivers (n = 72)	t/χ2(P value)
Age (µ ± SD)	36.34 ± 13.13	37.90 ± 13.83	33.17 ± 11.01	2.53 (0.112)
Sex				
Male	136 (62.39)	81 (55.48)	55 (76.39)	**8.98 (0.003)**
Female	82 (37.61)	65 (44.52)	17 (23.61)	
Marital status				
Single	73 (33.49)	34 (23.29)	39 (54.17)	**23.71 (< 0.001)**
Married/cohabiting	88 (40.37)	66 (45.21)	22 (30.56)	
Separated/divorced	45 (20.64)	34 (23.29)	11 (15.28)	
Widowed	12 (5.50)	12 (8.22)	0	
Employment				
Employed	132 (60.55)	80 (54.79)	52 (72.22)	**6.13 (0.013)**
Unemployed	86 (39.45)	66 (45.21)	20 (27.78)	
Area of residence				
Urban	60 (27.52)	51 (34.93)	9 (12.50)	**12.16 (< 0.001)**
Rural	158 (72.48)	95 (65.07)	63 (87.50)	
Level of education				
None	13 (5.96)	13 (8.90)	0	**12.99 (0.005)**
Primary	104 (47.71	63 (43.15)	41 (56.94)	
Secondary	59 (27.06)	36 (24.66)	23 (31.94)	
Post-secondary	42 (19.27)	34 (23.29)	8 (11.11)	
Number of children (µ ± *SD*)	2.59 ± 2.78	2.90 ± 2.87	1.96 ± 2.48	**2.39 (0.018)**
Type of housing				
Private	109 (50.00)	98 (67.12)	11 (15.28)	**109.90 (< 0.001)**
Public	49 (22.48)	3 (2.05)	46 (63.89)	
Rental	54 (24.77)	41 (28.08)	13 (18.06)	
Homeless	6 (2.75)	4 (2.74)	2 (2.78)	
Poverty level^a^				
Below poverty line	91 (41.74)	55 (37.67)	36 (50.00)	3.01 (0.083)
Above poverty line	127 (58.26)	91 (62.33)	36 (50.00)	

Statistically significant values are in bold (*p* < 0.05)

^a^Income level based on the national average poverty line of Uganda Shillings 16,643 per person per month[30]

**Table 2:** Clinical characteristics of the participants

Variable	N	With caregivers (n = 146)	Without caregivers (n = 72)	t/χ2(P value)
Mode of admission				
Voluntary	96 (44.04)	67 (45.89)	29 (40.28)	0.41 (0.523)
Involuntary	122 (55.96)	79 (54.11)	43 (59.72)	
History of previous admission in a mental health facility				
No	96 (44.04)	67 (45.89)	29(40.28)	0.62 (0.432)
Yes	122 (55.96)	71 (54.11)	43 (59.72)	
Years with mental illness (µ ± *SD*)	3.71 ± 6.41	4.27 ± 7.04	2.57 ± 3.41	1.85 (0.065)
Number of admissions (µ ± *SD*)	3.24 ± 7.00	3.69 ± 8.31	2.33 ± 2.68	1.35 (0.178)
Number of years since last admission (µ ± *SD*)	0.97 ± 2.40	0.93 ± 2.68	1.04 ± 1.72	−0.32 (0.751)
Frequency of follow-up of previous OPD appointment				
Regular	33 (15.14)	22(30.14)	8(18.60)	4.21 (0.240)
Sometimes	42 (19.27)	24(32.88)	14(32.56)	
Never	51 (23.39)	27(36.99)	21(48.84)	
Not applicable	92 (42.20)	65 (44.52)	27 (37.50)	
History of attempted suicide				
No	136 (62.39)	90 (61.64)	46 (63.89)	0.10 (0.748)
Yes	82 (37.61)	56 (38.36)	26 (36.11)	
History of being put in seclusion room during the admission				
No	183 (83.94)	120 (82.19)	63 (87.50)	1.01 (0.315)
Yes	35 (16.06)	26 (17.81)	9 (12.50)	
Money spent during this admission/10,000 (µ ± *SD*)	38.07 ± 59.40	20.23 ± 53.11	74.22 ± 55.18	−**6.97 (< 0.001)**
Patient is the Family bread winner				
No	128 (58.72)	82 (56.16)	46 (63.89)	1.19 (0.276)
Yes	90 (41.28)	64 (43.84)	26 (36.11)	
Comorbidities^b^				
No	139 (63.76)	100 (68.49)	39 (54.17)	**4.28 (0.038)**
Yes	79 (36.24)	46 (31.51)	33 (45.83)	
Diagnosis				
Schizophrenia and other primary psychosis (6A2)				
No	170 (77.98)	112 (76.71)	58 (80.56)	0.41 (0.520)
Yes	48 (22.02)	34 (23.29)	14 (19.44)	
Mood disorders (6A8)				
No	124 (56.88)	82 (56.16)	42 (58.33)	0.09 (0.761)
Yes	94 (43.12)	64 (43.84)	30 (41.67)	
Disorder due to substance use (6C4) and addictive behaviours (6C5)				
No	154 (70.64)	109 (74.66)	45 (62.50)	3.44 (0.064)
Yes	64 (29.36)	37 (25.34)	27 (37.50)	
Other diagnosis				
Yes	192 (88.07)	129 (88.36)	63 (87.50)	0.03 (0.854)
No	26 (11.93)	17 (11.64)	9 (12.50)	
Patient reviewed by a consultation liaison team				
No	205 (94.04)	139 (95.21)	66 (91.67)	1.08 (0.299)
Yes	13 (5.96)	7 (4.79)	6 (8.33)	
Side effects				
No	162 (74.31)	92 (63.01)	70 (97.22)	**29.56 (< 0.001)**
Yes	56 (25.69)	54 (36.99)	2 (2.78)	

Statistically significant values are in bold (*p* < 0.05)

^b^Comorbidities are other diseases not particularly mental illness such as diabetes, hypertension, among others existing with mental illness

### Length of hospital stay

**Fig. 2:**
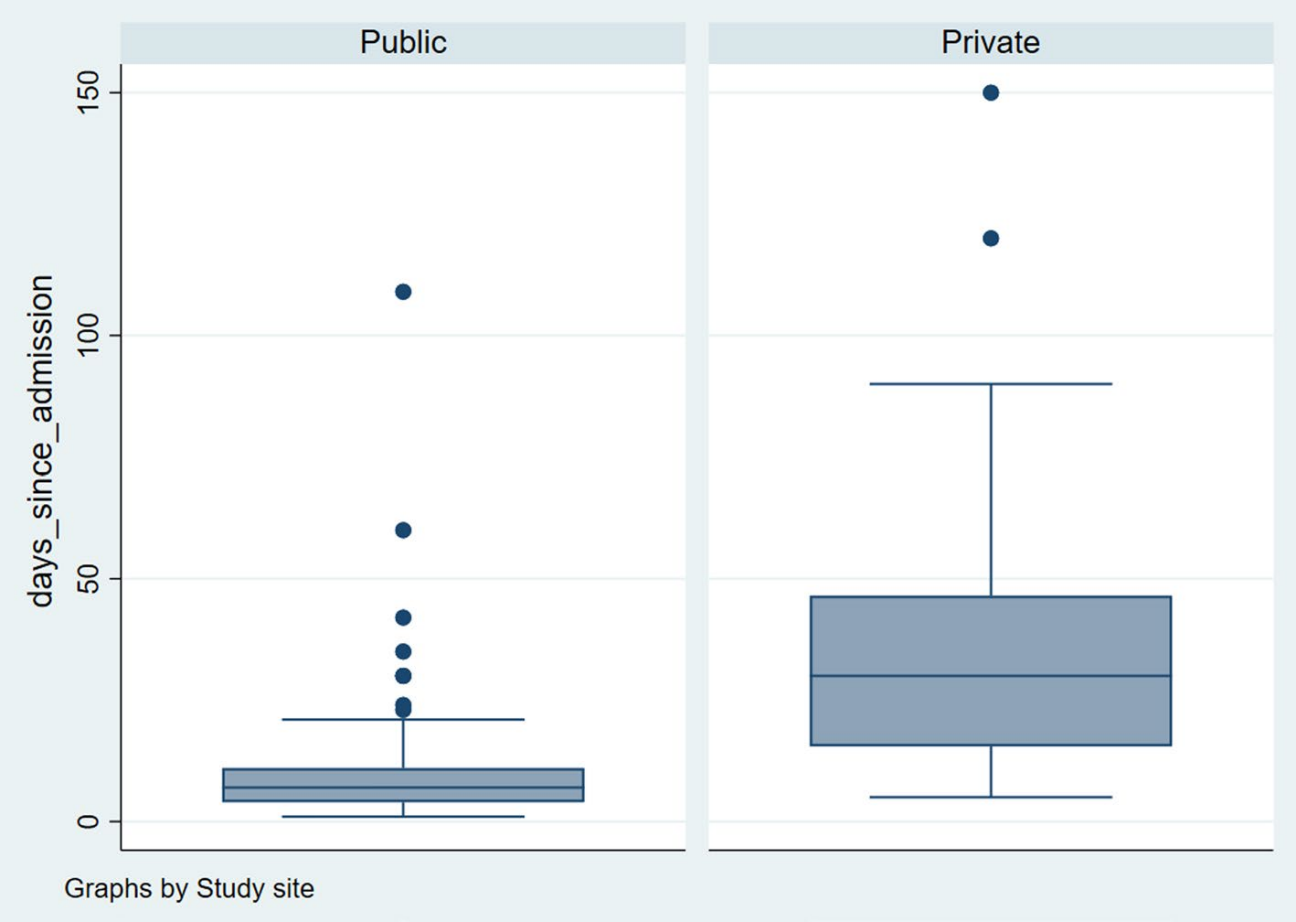
Box Plot showing the distribution of length of hospital stay by site

The average LoS in the two hospitals was 18.3 ± 22.3 days (36.4 in a hospital without caregivers and 9.38 in a hospital with caregivers). The median LoS in the hospital was higher among those who were admitted without caregivers compared to those with caregivers (30(30) vs. 7(7); χ^2^ = 68.95, *p* < 0.001) and the distribution of the LoS was statistically different in the two samples (*z* = −9.72, *p* < 0.001). The outliers were dropped from each site (above 93 days for private and 21.5 days for public), leaving us with a sample of 208 (138 public and 70 private). Details of the distribution of the LoS are shown in Fig. 2. The median LoS remained statistically significantly longer among participants without caregivers after removing outliers (6(4) vs. 30(30) days, χ^2^ = 75.30, *p* < 0.001).

### Factors associated with length of stay

At bivariate analysis, having no caregivers, younger age, married or widowed marital status, rural patient residence, fewer number of children, staying in a public type of housing, earning income above the poverty line, patient being a breadwinner for their family, increase in money spent during the hospital stay, schizophrenia diagnosis, and having received a review by the consultation-liaison team; were associated with LoS and were tested for collinearity and all the factors had a VIF below three and mean VIF of 1.54. At multivariable analysis, the LoS increased by almost 15 days when a person was admitted to a hospital without caregivers, over 10 days if an individual had a diagnosis of schizophrenia and about 8 days for individuals who were separated or divorced. However, for each dollar spent, the days increased slightly (less than a day), i.e., 0.14. No identified factor was associated with a reduction in LoS. This statistically significant final model was explained by 55.91% of the variation in LoS, (*p* < 0,001) (Table 3).

**Table 3:** linear regression analysis for factors associated with length of hospital stay

Variable	Total sample				With caregivers				Without caregivers			
	Univariable analysis		Multivariable analysis		Univariable analysis		Multivariable analysis		Univariable analysis		Multivariable analysis	
	Coefficient (95% confidence interval)	*P*-value	Adjusted coefficient (95% confidence interval)	*P*-value	Coefficient (95% confidence interval)	*P*-value	Adjusted coefficient (95% confidence interval)	*P*-value	Coefficient (95% confidence interval)	*P*-value	Adjusted coefficient (95% confidence interval)	*P*-value
Caregivers’ status												
With caregivers	1		1									
Without caregivers	27.01 (21.81–32.21)	** < 0.001**	14.88 (7.98–21.79)	** < 0.001**								
Age	− 0.27 (− 0.49–0.04)	** < 0.020**	− 0.13 (−0.39–0.13)	0.319	− 0.03 (− 0.09–0.02)	0.276			0.28 (−0.75–0.19)	0.245		
Sex												
Male	1				1				1		1	
Female	1.32 (− 4.83–7.47)	0.673			0.94 (−0.63–2.50)	0.239			12.30 (0.11–24.49)	**0.048**	2.68 (−6.96–12.32)	0.581
Marital status												
Single	1		1		1				1			
Married/cohabiting	−9.60 (−16.41—−2.79)	**0.006**	0.21 (−6.45–6.87)	0.951	0.70 (−1.30–2.70)	0.487			−7.22 (−18.66–4.21)	0.212		
Separated/divorced	−0.63 (−8.78–7.53)	0.880	7.68 (1.09–14.27)	**0.023**	0.44 (−1.86–2.73)	0..487			11.97 (−3.20–27.14)	0.120		
Widowed	−15.14 (−28.54—−1.73)	**0.027**	3.21 (−8.68–15.10)	0.595	1.16 (−1.95–4.27)	0.464			Omitted			
Employment												
Employed	1				1				1			
Unemployed	2.23 (−3.86–8.33)	0.471			0.74 (−0.84−2.31)	0.348			9.76 (−1.85–21.37)	0.098		
Area of residence												
Urban	1 (reference)		1		1		1		1			
Rural	11.84 (5.35–18.32)	** < 0.001**	4.48 (−0.65–9.60)	0.086	2.17 (0.59–3.74)	**0.007**	1.49 (−0.01–2.98)	0.050	9.96 (−5.59–25.52)	0.206		
Level of education												
None	1				1				1		1	
Primary	5.10 (−7.61–17.80)	0.430			−1.64 (−3.84–0.56)	0.143			−8.37 (−22.39–5.64)	0.237	−6.01 (−16.08–4.05)	0.237
Secondary	3.46 (−9.77–16.70)	0.607			−1.60 (−5.04–1.84)	0.359			−5.02 (−20.08–10.03)	0.508	−2.37 (−13.61–8.86)	0.674
Post−secondary	−7.55 (−21.26–6.16)	0.279			−1.70 (−3.68–0.27)	0.090			−21.00 (−37.49–−4.51)	**0.013**	−14.59 (−26.51–−2.68)	**0.017**
Number of children (µ ± *SD*)	−1.08 (−2.15–−0.02)	**0.046**	0.28 (−0.99–1.56)	0.663	0.08 (−0.18–0.35)	0.543			−0.70 (−2.81–1.41)	0.510		
Type of housing												
Private	1		1		1				1			
Public	28.64 (22.12–35.16)	** < 0.001**	7.55 (−0.01–15.11)	0.050	−4.12 (−9.42–1.17)	0.126			11.29 (−3.22–25.81)	0.125		
Rental	5.25 (−1.06–11.56)	0.102	4.90 (−0.54–10.35)	0.078	−0.43 (−2.18–1.32)	0.628			0.92 (−16.7 –18.55)	0.918		
Homeless	14.68 (−1.23–30.58)	0.070	3.74 (−9.48–16.97)	0.578	3.79 (−0.82–8.40)	0.106			26.45 (−6.63–59.55)	0.115		
Poverty level^a^												
Below poverty line	1		1		1		1		1		1	
Above poverty line	−12.05 (−17.88—−6.22)	** < 0.001**	−2.50 (−7.37–2.38)	0.314	−1.94 (−3.53 –−0.34)	**0.018**	−1.24 (−2.88–0.40)	0.136	−11.87 (−22.02–−1.72)	**0.023**	2.61 (−6.51–11.73)	0.569
Patient the bread winner in their family?												
No	1		1		1		1		1		1	
Yes	−9.74 (−16.66—−3.83)	** < 0.001**	−3.96 (−8.86–0.93)	0.112	−1.69 (−3.22–−0.15)	**0.031**	−0.83 (−2.36 –0.70)	0.285	−12.66 (−23.12–−2.19)	**0.019**	−7.35 (−16.14–1.45)	0.100
Money spent during this admission	0.21 (0.17–0.25)	** < 0.001**	0.14 (0.09–0.18)	** < 0.001**	0.01 (−0.03–0.02)	0.922			0.37 (0.27 –0.46)	** < 0.001**	0.34 (0.25–0.44)	** < 0.001**
Comorbidities^b^												
No	1				1				1			
Yes	0.76 (−5.44–6.96)	0.809			0.04 (−1.61–1.69)	0.959			−4.62 (−15.14–5.90)	0.384		
Schizophrenia and other primary psychosis (6A2)												
No	1		1		1				1		**1**	
Yes	10.70 (3.65–17.75)	**0.003**	10.68 (5.53–15.83)	** < 0.001**	0.78 (−1.08–2.64)	0.408			15.60 (2.14–29.07)	**0.024**	10.79 (0.75–20.84)	**0.036**
Mood disorders (6A8)												
No	1				1		1		1			
Yes	−1.87 (−7.89–4.14)	0.541			2.57 (1.06–4.08)	**0.001**	1.49 (−0.3–3.02)	0.055	−2.05 (−12.69–8.59)	0.702		
Disorder due to substance use (6C4) and addictive behaviours (6C5)												
No	1				1		1		1			
Yes	−1.06 (−7.61–5.48)	0.749			−2.49 (−4.20–−0.78)	**0.005**	−1.13 (−2.85–0.60)	0.199	0.70 (−10.13–11.52)	0.898		
Other diagnosis												
No	1				1				1			
Yes	−4.49 (−13.67–4.69)	0.336			−0.41 (−2.91–2.09)	0.748			−12.64 (−28.08–2.80)	0.107		
Patient reviewed by a consultation liaison team												
No	1		1		1				1			
Yes	12.68 (0.21–25.16)	**0.046**	−0.86 (−10.07–8.34)	0.854	1.28 (−2.88–5.44)	0.543			15.23 (−3.23–33.69)	0.104		
Side effects												
No	1				1		1		1			
Yes	−6.17 (−12.94–0.61)	0.074			2.76 (1.20–4.31)	**0.001**	2.29 (0.78–3.80)	**0.003**	−13.77 (−58.05–30.52)	0.537		

Statistically significant values are in bold (*p* < 0.05)

^a^Income level based on the national average poverty line of Uganda Shillings 16,643 per person per month[30]

^b^Comorbidities are other diseases not particularly mental illness such as diabetes, hypertension, among others existing with mental illness

In a hospital with caregivers, the factors associated with the increase in LoS were area of residence, diagnosis of bipolar affective disorder, and having side effects for psychoactive medications. However, earning monthly income above the poverty line, being the breadwinner, and having a diagnosis of substance use reduced the LoS. These were tested for collinearity; all the factors had a VIF below three and the mean VIF was 1.15. At multivariate regression analysis, only having side effects to psychoactive medications statistically significantly increased the length of hospital stay (aCoef: 2.29; 95 CI 0.78–3.80; *p* = 0.003) (Table 3).

In a hospital without caregivers, the following factors were associated with an increase in the LoS at bivariate analysis: female gender, money spent during admission, and having a diagnosis of schizophrenia. However, having highest level of education as post-secondary, earning monthly income above the poverty line, and being the family breadwinner reduced LoS. These were tested for collinearity and all the factors had a VIF below three and mean VIF of 1.23. At multivariate analysis, the following reduced the LoS, money spent during hospital stay (aCoef: 0.34; 95% CI 0.25 – 0.44; *p* < 0.001) and a diagnosis of schizophrenia (aCoef: 10.79; 95% CI 0.75 – 20.84; *p* = 0.036). However, having post-secondary level of education reduced the LoS (aCoef: − 14.59; 95% CI − 26.51– −2.68; *p* = 0.017) (Table 3).

## Discussion

This study reports on length of hospital stay (LoS) and its associated factors among patients admitted in two hospitals in Uganda, one with patients admitted with caregivers (public) and another without caregivers (private). The average LoS was 18.30 ± 22.28 days.

The LoS was shorter based on the recommended cutoff of 28 days for longer LoS as documented in the literature [31, 32]. However, the reported average LoS is similar to previously reported Ugandan ranges of 2 to 3 weeks in 2014 [13]. This is due to both findings coming from the same country whose policies about mental illness have not metamorphosized much [13, 24]. However, the overall average LoS found in our study was shorter than what has been reported in other sub-Saharan mental health facilities, i.e., Malawi (22 days), Nigeria (25 – 28.7 days), and Ethiopia (28.7 days) [3, 10–12]. This may be attributed to the LoS having data from a hospital with caregivers involved in patient care, which is associated with a significant reduction in LoS [19, 22, 23]. The LoS observed in our study is longer than the ten days reported in USA [6, 8]. This may be due to the advancement in the technology in the USA to manage patients with mental illness, such as the use of ECT, deep brain stimulation, and other new modalities that improve the chances of early recovery, thus early discharge [3]. In addition, the USA and other high-income countries' mental health systems are more developed with better community psychiatry programs, a higher number of human resources, better funding, and the presence of insurance policies and programs that may influence patient discharge [33]. The average LoS was still lower than many developed countries with advanced technology, such as Chile (30.5 days), Czech Republic (41.2 days), Hungary (32.6 days), Israel (52.4 days), Poland (35.6 days), Slovenia (34.2 days), Spain (36.3 days), United Kingdom (36.5 days), and Korea (168) [1]. This may be due to the different forms and stages of deinstitutionalization in these countries, such as the use of group homes and trust homes, among others, and some caregivers in these countries prefer their patients to stay in long-term institutionalization [34]. In addition, the lack of caregiver involvement may be an integral factor in the increased LoS in these countries since most of the care of people with mental health is left to mental health institutions.

In our study, caregiver’s presence during admission was associated with a shorter LoS. Not having caregivers during admission increased patient stay by more than 18 days. This is attributed to the roles performed by the caregivers as reported by other researchers [19, 22, 23]. The different roles such as emotional support, encouraging and motivating the patient, assistance with practical tasks like managing finances, transportation, feeding, and other ADL, for example, personal hygiene, and a decision on whether a patient needs admission or discharge influence patient’s recovery, hence a shorter LoS [19, 22, 23]. The findings are similar to those from a study done in 2007 in Austria among caregivers of elderly persons with mental illness that found caregiver’s presence and roles associated with reduced LoS [35]. The findings also echoed findings of other studies among mental patients with caregivers [11, 36, 37]. With such a large significant difference in the LoS, despite multiple similarities between the hospital settings with caregivers and without caregivers, all mental health facilities should encourage caregiver presence during patient admission for better patient care. The reasons for the difference may be due to the presence of caregivers and the pressure in the hospital systems to admit. The public hospital (with caregiver) compared to the private hospital (without caregiver) may have many admissions due to having little cost attached to patient stay in this low-income country (Uganda); as a result, they may end up discharging patients early to provide space for new admissions. The other reasons for the difference leading to caregivers’ presence being associated with LoS could have been due to the private hospital being in a more rural setting where patients are known to report late for psychiatry care thus reaching the facility when they are more severe symptoms, thus, they may require longer days for treatment – LoS.

Having a diagnosis of schizophrenia, especially among patients without caregivers, was associated with increased LoS. A diagnosis of schizophrenia has consistently been associated with the longest LoS among patients with mental illness [10, 12, 14, 20, 38, 39]. This is mainly attributed to the severity of the illness and its impact on the perception that hinders individual’s functionality outside institutions, such as an increase in criminality [38, 40]. Schizophrenia has also been associated with multiple complications such as fractures, malnutrition, and other co-infections (39), all of which may take longer to recover, hence increasing the LoS. The longer LoS among patients with schizophrenia may also be linked to the severe neurocognitive function impairment among patients with schizophrenia that deems them unfunctional outside the hospital environment. Moreover, cognitive impairment—a common feature in schizophrenia, might hinder the treatment of comorbidities such as fractures, diabetes, hypertension, HIV, among others, due to the inability to follow instructions [35, 39]. The cognitive deficit is worsened by antipsychotics, which are the main treatment for schizophrenia, thus worsening decision-making and prolonging recovery. This may be worse for patients in low-income settings like Uganda, where typical antipsychotics (associated with severe cognitive impairment), are the mainstay of treatment, and yet patients with schizophrenia receive higher doses which may contribute to staying longer in hospitals.

As reported by other studies, marital status is a significant influencer of the LoS [11, 41, 42], and in our study, being separated or divorced increased the LoS. These findings are supported by Adegunloye et al. (2009), who found a dysfunctional social network between partners that usually lead to divorce and separation may lead to longer durations of untreated psychosis; thus, more severe symptoms of prolonged LoS [11]. On the other hand, marriage significantly reduces the LoS, mainly attributed to the positive impact of social support to the significant other about problems expressed; motivation and encouragement to meet goals; assistance with drug monitoring and reminders of appointments; and opportunities for relaxation and memory sharing [11]. In addition, partners of married patients may be more interested in the disease presentation and management, enhancing early admission hence shortening the symptoms, less severity, and shorter LoS [11]. In most settings, the longer patients stay admitted, the more the expenses, especially in a private hospital (without caregivers). This was not surprising since many studies have found an association between cost of care and length of stay [5, 8, 43].

### Study strength and limitations

The major strength of this study is that it is a cross-sectional study involving data collection from the participants, contrary to most of the studies about LoS, which are commonly retrospective in design. The study also had suitable comparison groups to assess the impact of caregiver presence during a mental patient hospital stay. However, this study's findings should be interpreted with caution due to the following limitation. (1) It was a cross-sectional study, and the causality of increased LoS could not be determined, whereas more robust methodological studies are highly suggested. (2) We recruited patients from only two hospitals in the country, and they may not be representative of the characteristics of all mental health service centers in the country. (3) Despite information obtained from patients at discharge, this may not be reliable since most patients still experience reality distortion within the first few days following discharge. (4) We did not include patients with communication disorders who usually stay longer in hospitals, thus skewing the LOS. (5) Despite the two hospitals being very similar, KIUTH is a private hospital (it does not receive enough from the government to reduce its expenditure) and patients may stay longer due to the failure of individuals to pay the high hospital-related costs on time. Lastly, recall bias was possible, especially with items such as money spent during the admission since most patients were experiencing severe mental illness.

## Conclusion

Mentally ill patients in southwestern Uganda have a shorter LoS (below 28 days), and the stay is much shorter among patients with caregivers compared to those without. Therefore, we recommend caregivers' presence during patients’ hospital stay to reduce the LoS. In addition, patients diagnosed with schizophrenia should be managed more with community psychiatry approaches such as assertive community teams to minimize complications associated with longer LoS.

## Data Availability

The datasets used and/or analysed during the current study are available from the corresponding author on reasonable request.

## Abbreviations

ADL: Activities of Daily Living
BAD: Bipolar affective disorder
BNRH: Butabika National referral Hospital
HIV: Human immunodeficiency virus
IQR: Inter quartile range
KIUTH: Kampala International University Teaching Hospital
LoS: Length of stay
MRRH: Mbarara Regional Referral Hospital
VIF: Variance Inflation Factor
